# Persistent IFN-I signaling associated with the HIV-1 reservoir fuels immune exhaustion and reveals therapeutic targets

**DOI:** 10.3389/fimmu.2026.1829548

**Published:** 2026-08-25

**Authors:** Zhihui Zhang, Pengfei Ren, Meng Deng, Qiong Li, Zhaoyun Chen

**Affiliations:** Henan Clinical Research Center of Infectious Diseases (AIDS), The Sixth People’s Hospital of Zhengzhou, Zhengzhou, China

**Keywords:** HIV-1 reservoir, immune checkpoint, immune exhaustion, type I interferon signaling pathway, therapeutic targets

## Abstract

The viral reservoir established after HIV-1 infection remains the primary barrier to curing HIV. Recent studies suggest that proviral HIV-1 DNA, low-level viral transcripts, and other reservoir-derived viral products may contribute to persistent innate immune activation and type I interferon (IFN-I)-related inflammatory programs. This sustained activation drives a state of chronic immune activation, which induces and maintains exhaustion in immune cells such as CD4^+^ T cells. Exhaustion is characterized by loss of effector function and upregulation of inhibitory receptors (immune checkpoints), severely compromising the host’s antiviral immune response. In this review, we systematically examine the molecular and cellular mechanisms by which the HIV-1 reservoir fuels immune exhaustion through the IFN-I signaling pathway. We further explore potential intervention targets along this pathway, providing a theoretical framework for the development of novel therapeutic strategies aimed at reservoir elimination or reversal of immune exhaustion.

## 1 Introduction

Following infection with Human Immunodeficiency Virus Type 1 (HIV-1), Antiretroviral Therapy (ART) effectively suppresses viral replication. Nevertheless, integrated proviral DNA persists in resting CD4^+^ T cells and other cellular reservoirs, establishing a stable latent pool that remains the principal obstacle to a cure (1). Even during long-term ART, low-level “leaky” transcription from these reservoirs continuously releases viral antigens, sustaining a chronic inflammatory environment defined as a persistent state of immune activation driven by continuous antigen exposure and characterized by upregulation of inhibitory receptors (e.g., KLRG1, TIGIT), imbalanced T-bet/Eomes expression, and persistent pro-inflammatory signaling – all key drivers of CD8^+^ T cells exhaustion (2–7).

Type I interferon (IFN-I) signaling plays a dual role in HIV-1 infection: while essential for early antiviral defense, its chronic activation during persistent infection promotes immune dysfunction and exhaustion (8, 9). Notably, sustained IFN-I signaling – even under conditions of viral suppression – impairs NK and ILC3 function and drives CD4^+^ T cells toward an exhausted phenotype (10, 11). Mechanistically, IFN-I signaling, predominantly driven by IFN-α subtypes in the peripheral blood, promotes the upregulation of multiple immune-checkpoint molecules, including PD-1 and TIGIT, on HIV-specific T cells through JAK-STAT-dependent transcriptional programs (2, 12, 13). This checkpoint upregulation contributes to a state of T cell exhaustion characterized by loss of effector function and impaired proliferative capacity – features that, while resembling those observed in cancer immunology, are increasingly recognized as having HIV-specific molecular determinants (14–16). A detailed understanding of the cascade linking reservoir-derived viral products, innate immune sensing, IFN-I signaling, and T cell dysfunction is therefore crucial for designing targeted interventions along this pathway (17).

In summary, IFN-I signaling exhibits a context-dependent role in HIV-1 infection: early engagement can suppress viral replication, whereas persistent signaling – fueled by the latent reservoir during chronic infection – exacerbates immune pathology (8, 9). This review systematically outlines this mechanistic link and focuses on potential therapeutic strategies that target key nodes within the IFN-I pathway, with the aim of decoupling antiviral efficacy from pathological immune activation. By doing so, it aims to provide new insights for the design of “functional cure” or immune-reconstitution strategies (18).

## 2 HIV-1 reservoir-driven chronic IFN-I signaling: from persistent activation to functional switch

### 2.1 Persistent antigen exposure from the reservoir and innate immune sensing

Although antiretroviral therapy (ART) effectively suppresses HIV-1 replication, integrated proviral DNA in the host genome—particularly within resting memory CD4^+^ T cells—constitutes a stable latent reservoir, representing the principal obstacle to a cure (1, 19). This latent state is not entirely quiescent; studies indicate that even in the absence of detectable plasma viral load, proviruses within the reservoir undergo spontaneous, intermittent, or induced low-level transcription, a phenomenon referred to as “leaky” transcription (20). This low-level transcription can produce spliced viral mRNA, unspliced genomic RNA, and limited viral proteins such as Nef and Tat (1). For instance, using flow cytometry-fluorescence *in situ* hybridization (flow-FISH), CD4^+^ T cells expressing abortive or productive HIV-1 transcripts have been directly detected in the peripheral blood mononuclear cells (PBMCs) of ART-treated individuals without activation, confirming the ongoing low-level transcriptional activity of the viral reservoir *in vivo (*1, 21).This activity is closely related to the size and heterogeneity of the viral reservoir (19). Moreover, reservoir burden and immune activation markers correlate in ART-treated cohorts, but direct causal linkage to IFN-I signatures remains incompletely established (22). There may be differences in the size and latency reversal characteristics of viral reservoirs among different HIV-1 subtypes, suggesting that intrinsic viral factors also influence the dynamics of transcription during latency (20). The persistent presence of viral nucleic acids and protein products, even in limited quantities, may provide an intermittent or low-level source of immune stimulation for the immune system, potentially driving chronic immune activation and exhaustion.

During residual replication, abortive infection, or episodes of latency reversal, viral DNA intermediates and viral RNA species may be exposed to cytosolic or endosomal sensors and may constitute pathogen-associated molecular patterns (PAMPs) that can be detected by host pattern recognition receptors (PRRs), thereby initiating a sustained innate immune response (23). Among these PRRs, cyclic GMP-AMP synthase (cGAS) is a key sensor for cytosolic DNA. Research has shown that early in infection, the HIV-1 capsid protein is recognized by the host factor PQBP1, which subsequently recruits cGAS to the viral capsid. This “two-step verification” strategy enables the innate immune surveillance mechanism to selectively respond to low-abundance HIV-1 DNA PAMPs (24, 25). Furthermore, viral RNA can be recognized by RNA sensors such as RIG-I and MDA5 (23). Activation of these sensors leads to the initiation of downstream signaling pathways, ultimately promoting the activation of IFN regulatory factors IRF3 and IRF7, as well as NF-κB. Activated IRF3, IRF7, and NF-κB enter the nucleus, driving the sustained production of IFN-Is (IFN-α/β) and pro-inflammatory cytokines (23). Even in ART-treated individuals, this ongoing state of innate immune activation can still be detected in immune cells such as myeloid dendritic cells, and it is associated with the size of the reservoir (26, 27).

Notably, the PRR response is dynamically regulated over the course of infection: during acute HIV-1 infection, PRRs such as TLR7/8 and RIG-I are robustly upregulated in peripheral blood, driving rapid IFN-I production (23). In contrast, chronic infection is characterized by broad downregulation of TLR4 and TLR8 at mucosal sites, particularly in the gut, which is associated with dysbiosis and impaired barrier integrity (7, 28), suggesting that the innate sensing machinery shifts from a protective to a dysfunctional state as infection persists. Thus, the HIV-1 reservoir is not merely a viral “sanctuary” but also a continuous source of stimulation that, by persistently providing viral PAMPs, drives chronic inflammation and immune dysregulation. Having established that the reservoir continuously produces viral antigens and nucleic acids, we next examine how these components trigger and sustain chronic IFN-I signaling.

### 2.2 Establishment of chronic IFN-I signaling and its functional transformation

The persistent innate immune activation driven by the reservoir leads to the long-term, aberrant expression of IFN-I signaling, whose net effect may shift from predominantly antiviral during acute infection toward immunopathogenic and exhaustion-promoting during chronic infection. During the acute infection phase, the IFN-I response is crucial for limiting early viral dissemination and establishing adaptive immunity (9). For example, in women with acute/early infection, higher expression of IFN-I signaling pathway-related genes in CD4^+^ T cells correlates with lower observed viral loads, suggesting a contribution to early viral control (29).

However, during the chronic infection stage, the persistent presence of viral antigens leads to the long-term, low-level activation of IFN-I signaling. This state of chronic immune activation persists even after long-term ART achieves viral suppression (6, 10, 30). This chronic, as opposed to acute, IFN-I signaling alters its biological effects, directly promoting immune exhaustion. It induces a series of interferon-stimulated genes (ISGs), whose sustained high expression is a hallmark of chronic immune activation and dysfunction (7, 28). For instance, transcriptomic analyses show that ISGs are persistently upregulated in PBMCs of chronic HIV-infected individuals (28). While some ISG products (e.g., APOBEC3G, MX2) can directly inhibit viral replication, the persistent ISG environment also creates an immunosuppressive state. It can encode negative regulators such as USP18 and SOCS1, or molecules that mediate apoptosis, collectively weakening T cell proliferation and effector function (9, 31, 32). In humanized mouse models of HIV-1 infection, sustained IFN-I signaling drives NK cells to differentiate into a decidual NK cell-like phenotype with immunosuppressive characteristics, impairing their cytotoxic activity (10). Furthermore, epigenetic studies confirm that HIV infection-related differentially methylated CpG sites are enriched in genes related to IFN-α, inflammation, and apoptosis pathways, solidifying this immunosuppressive environment at the epigenetic level (33). Importantly, in advanced Acquired Immunodeficiency Syndrome (AIDS)—characterized by profound CD4^+^ T cell depletion and loss of lymphoid tissue architecture—the IFN-I response may shift further from a protective to a pathogenic mode, with dysregulated IFN-α/β production contributing to sustained immune activation and tissue damage (8, 9), although the precise mechanisms at this late stage remain incompletely understood. Therefore, by establishing a persistent ISG expression program, chronic IFN-I signaling constructs an extensive immunosuppressive network that, while attempting to control the virus, fundamentally transforms its function from protective antiviral to promoting immune dysfunction and exhaustion, setting the stage for the core mechanisms outlined in Part 2.

## 3 Core mechanisms of IFN-I signaling driving immune exhaustion

### 3.1 Regulation: induction of immune checkpoint molecules and metabolic reprogramming

IFN-I signaling can promote the expression of multiple inhibitory receptors, partly through JAK-STAT-dependent transcriptional programs and inflammatory secondary mediators (34, 35). In the context of HIV infection, exhausted CD8^+^ T cells express high levels of multiple checkpoint molecules, and their expression levels correlate positively with plasma IFN-α levels and reservoir size (13). This association is particularly evident in patients co-infected with HIV-1 and Mycobacterium tuberculosis, where the expression of TIM-3, CTLA-4, LAG-3, and PD-1 on both CD4^+^ and CD8^+^ T cells is significantly increased, negatively correlated with CD4^+^ T cell counts and positively correlated with plasma viral load, directly linking to disease progression (36). Beyond PD-1, other inhibitory checkpoints such as TIGIT and LAG-3 are also upregulated in HIV-specific T cells and contribute to exhaustion. TIGIT is highly expressed on CD8^+^ T cells in HIV-infected individuals and correlates with impaired cytotoxic capacity, while its co-expression with PD-1 is enriched on latently infected CD4^+^ T cells (12, 13). LAG-3, which binds MHC class II, synergizes with PD-1 to suppress T cell proliferation and effector functions, and its blockade has been shown to restore HIV-specific T cell responses in preclinical models (13, 37). These findings collectively argue for a multi-checkpoint blockade strategy rather than targeting PD-1 alone. These upregulated checkpoint molecules transmit strong inhibitory signals upon binding to their ligands, suppressing T cell receptor signaling, key cytokine production (e.g., IFN-γ), and cytotoxic functions, ultimately leading to T cell functional loss (38, 39). This IFN-I-associated increase in checkpoint molecule expression constitutes one of the core molecular mechanisms of immune exhaustion in chronic HIV-1 infection.

Furthermore, beyond directly suppressing T cell function via checkpoint upregulation, chronic IFN-I signaling stabilizes the exhaustion program at a deeper level by reshaping T cell metabolism and differentiation fate. Persistent IFN-I signaling profoundly alters T cell metabolic reprogramming: it perturbs mitochondrial fitness, oxidative phosphorylation, and glycolytic flexibility in a context-dependent manner, a metabolic shift closely linked to T cell functional exhaustion (40, 41). In the tumor microenvironment (TME), similar metabolic competition has been shown to impair CD8^+^ T cell function (42). In HIV infection, this metabolic dysregulation may impair the ability of T cells to maintain long-term functionality and memory formation. More importantly, IFN-I can promote CD8^+^ T cell differentiation into exhausted precursor cells or terminally exhausted T cells, while inhibiting differentiation into memory precursor cells with self-renewal capacity (43, 44). This shift in differentiation fate is regulated by key transcription factors: IFN-I epigenetically stabilizes the exhaustion program by influencing the expression of TOX and NR4A, making the exhausted state difficult to reverse even when ART effectively suppresses the virus (45). Research also indicates that mitochondrial dysfunction is a key feature of T cell exhaustion, and IFN-I signaling may exacerbate this process (43). Thus, chronic IFN-I signaling, by coordinately regulating metabolic pathways and transcriptional networks, not only acutely suppresses T cell function but also may durably alter their functional state, locking them into a durably stabilized exhausted state that is difficult, but not necessarily impossible, to reverse.

### 3.2 Indirect effects: disruption of lymphoid tissue structure and function

The impact of sustained IFN-I responses on the immune system extends beyond intrinsic cellular functional changes to the disruption of the microenvironment on which immune cells rely for survival and interaction—namely, lymphoid tissue structure and function. Chronic IFN-I signaling is closely associated with structural damage and fibrosis of lymphoid tissues such as lymph nodes and the spleen, severely affecting normal immune cell migration, intercellular interactions, and survival microenvironments (46). In the context of HIV-1 infection, this disruption is particularly pronounced. IFN-I can induce changes in high endothelial venules and activate fibroblastic reticular cells, thereby damaging germinal center structures (47). Germinal centers are critical sites for B-cell maturation and production of high-affinity antibodies; their structural damage directly impairs follicular helper T cell function, which is essential for maintaining germinal center responses and promoting B-cell production of virus-specific antibodies. Aberrant IFN-I signaling pathways, as seen in systemic lupus erythematosus, can lead to disrupted immune homeostasis and persistent biological effects, a phenomenon that may parallel the chronic immune activation observed in HIV infection (35). When germinal center structure and Tfh function are compromised, B-cell responses and virus-specific antibody production are severely affected, weakening humoral immunity’s ability to control the virus (47). Additionally, under chronic inflammatory conditions, tertiary lymphoid structures may form, though their functions may be dysregulated (48). In HIV infection, persistent viral antigen stimulation and IFN-I-driven inflammation may lead to abnormal lymphoid tissue remodeling, creating a microenvironment that neither effectively initiates new immune responses nor maintains normal immune surveillance. This destruction of tissue structure and function, combined with the T cell exhaustion and metabolic dysregulation directly driven by IFN-I, forms a self-reinforcing inflammatory loop that supports persistent viral existence and leads to a comprehensive decline in immune system function.

**Figure 1:**
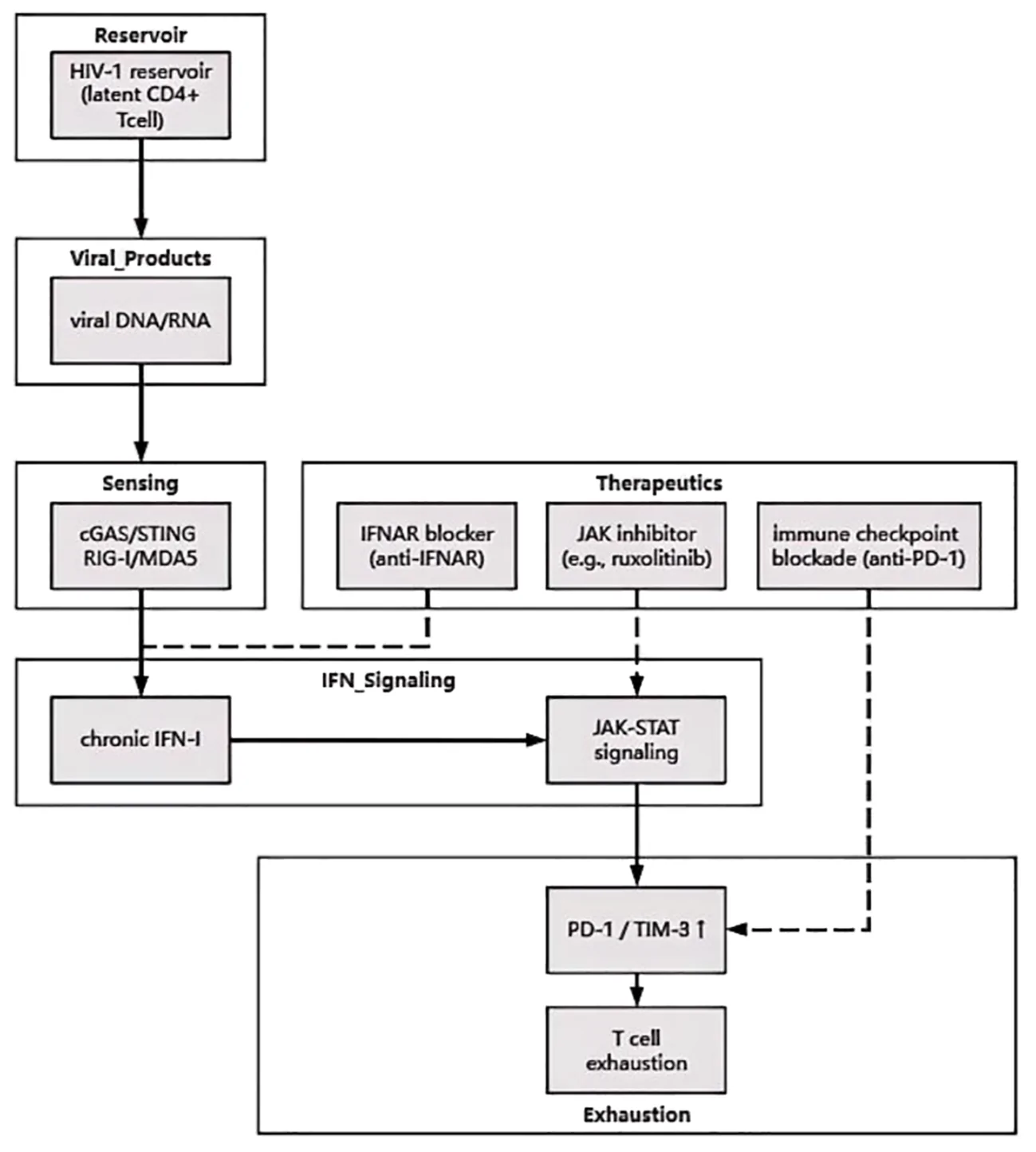
Schematic overview of the “reservoir-IFN-exhaustion” axis. The diagram illustrates the sequential cascade from the HIV-1 reservoir (latent CD4^+^ T cells) to persistent viral nucleic acids (DNA/RNA), innate sensing via cGAS/STING and RIG-I/MDA5, chronic type I interferon (IFN-I) production, JAK-STAT signaling, upregulation of immune checkpoint molecule PD-1/TIM-3, and ultimately T cell exhaustion. A therapeutic intervention node (JAK inhibitor) is indicated below the cascade. Arrows denote direction of signal flow.

The core signaling cascade from the HIV-1 reservoir to T cell exhaustion, along with a key therapeutic intervention node, is schematically summarized in **Figure 1**.

## 4 HIV-1 reservoir - IFN axis: clinical and experimental evidence

### 4.1 Clinical evidence linking reservoir burden to IFN-I signatures and exhaustion

Clinical studies provide evidence for the association between the reservoir-IFN axis and immune exhaustion. In ART-treated individuals, the persistent HIV-1 reservoir sustains chronic IFN-I signaling despite undetectable plasma viremia. ISG expression correlates with HIV-1 infection in peripheral compartments during acute and chronic infection, indicating an ongoing IFN-I transcriptional signature driven by the reservoir (49). Soluble immune activation markers including sCD14, sCD163, IL-6, and IP-10 have been linked to HIV-1 DNA levels in longitudinal reservoir studies, with integrated HIV-1 DNA load positively associated with elevated sCD14 (50, 51). IP-10 further promotes latent HIV infection in resting memory CD4^+^ T cells via the LIMK-cofilin pathway, reinforcing the role of this chemokine in reservoir persistence (52). These clinical observations demonstrate that reservoir burden correlates with chronic IFN-I-driven innate immune activation.

This persistent immune activation is closely tied to T cell exhaustion and immune dysfunction. In ART-suppressed individuals, PD-1hi T cells persist and correlate positively with HIV DNA levels, while PD-1 expression on HIV-specific CD8^+^ T cells is associated with higher viral loads, lower CD4^+^ T cell counts, and impaired effector function (15, 16). A higher proportion of PD-1+ HIV-specific CD8^+^ T cells before ART initiation strongly correlates with HIV DNA at one year post-ART, indicating that early CD8^+^ T cell exhaustion predicts post-treatment viral persistence (16). Collectively, these clinical data establish a correlative chain from viral persistence to chronic IFN-I stimulation and subsequent T cell exhaustion, identifying the reservoir burden and its provoked IFN-I signaling as key therapeutic targets.

### 4.2 Experimental evidence supporting the reservoir-IFN-exhaustion axis

Direct mechanistic evidence linking viral products to IFN-I-driven immune suppression comes from co-culture studies. Svanberg and colleagues showed that HIV-1-derived oligonucleotides activate the STING pathway in dendritic cells, leading to type I IFN production and subsequent suppression of T cell proliferation and function. Importantly, this suppression could be reversed by blocking the IFNAR1 receptor, establishing a causal chain from viral nucleic acids to IFN-I signaling and immune dysfunction (30). In humanized mouse models of chronic HIV-1 infection under suppressive ART, persistent IFN-I signaling was shown to impair innate lymphoid cell function and drive CD4^+^ T cells toward an exhausted phenotype, further supporting the pathogenic role of sustained IFN-I (10). Additionally, studies in SIV-infected macaques on ART revealed persistent activation of monocytes and macrophages along with cell senescence signatures, indicating that innate immune dysregulation persists despite virologic suppression (28). Proof-of-concept for targeting this axis has been provided by intervention studies. Treatment with the autophagy inducer rapamycin reduced IFN-I-mediated inflammation and enhanced anti-HIV-1 T cell responses *in vivo (*11). Collectively, the combination of co-culture, humanized mouse, and SIV models validates that the reservoir-driven IFN-I axis is operative and can be modulated. These findings establish a tight mechanistic link between the reservoir, chronic IFN stimulation, and immune exhaustion, supporting further preclinical evaluation for developing novel interventions targeting the “reservoir-IFN axis”.

### 4.3 From validation to intervention: identifying targetable nodes

The clinical and experimental evidence described above not only validates the central role of the reservoir-IFN axis in driving immune exhaustion but also clearly reveals a series of key nodes amenable to intervention along this pathway, including viral sensors (e.g., cGAS, STING), the IFN-I receptor (IFNAR), and downstream signaling molecules (e.g., JAK, STAT). Modulating these nodes offers new perspectives for optimizing current major HIV cure strategies.

For instance, in the “shock and kill” strategy, latency-reversing agents aim to wake up the latent virus. However, the concomitant activation of IFN-I signaling could potentially exacerbate immune exhaustion, thereby compromising the “kill” phase. Consequently, combining an IFN signaling inhibitor (e.g., a JAK inhibitor) might mitigate the upregulation of checkpoint molecules while preserving the reversal effect, thus enhancing clearance efficiency. Conversely, in the “block and lock” strategy, certain compounds designed to push the virus into a deeper state of latency may have their pro-latency function modulated by IFN signaling. Therefore, systematically integrating the “reservoir-IFN axis” mechanism elaborated in this review into the design of cure strategies holds promise for achieving more effective combinatorial therapies.

The established evidence and therapeutic implications set the stage for a detailed evaluation of treatment strategies targeting each node of this axis in the following section.

## 5 Therapeutic interventions targeting IFN-I signaling to reverse immune exhaustion

### 5.1 Directly regulating the IFN signaling

Directly regulating IFN-I signaling is a key strategy for reversing immune exhaustion in HIV-1 infection. Given that chronic IFN-I signaling is a potential contributor of immune activation and dysfunction, blocking its initiation or downstream pathways represents a promising therapeutic direction.

Anifrolumab has demonstrated clinical efficacy in systemic lupus erythematosus, but its application to HIV-associated chronic IFN-I activation remains hypothetical and requires preclinical validation (53). Evidence from HIV-related *in vitro* systems suggests that IFNAR blockade can reverse some IFN-I-mediated suppressive effects, supporting further evaluation of this strategy (30). In SIV models or early clinical trials, such blockade aims to mitigate the adverse effects of chronic IFN-I signaling, thereby improving overall immune function. For example, enhanced STAT1 phosphorylation but impaired transcriptional upregulation has been observed in severe COVID-19 patients, indicating an imbalance in JAK/STAT signaling. This suggests that direct intervention in IFNAR signaling may help restore normal immune cell function (54). Furthermore, JAK inhibitors provide a mechanistic rationale for dampening IFN-driven inflammatory signaling, but direct evidence that they reverse PD-1-mediated exhaustion in ART-treated HIV infection remains limited (55, 56). JAK inhibitors have also been shown to alleviate IFN-γ-driven inflammatory cascades in diseases such as inflammatory bowel disease by interfering with signaling from multiple cytokines, including IFN-γ, providing a rationale for modulating excessive immune responses in the context of HIV infection (57).

Another refined regulatory strategy involves enhancing negative feedback pathways by augmenting the activity of negative feedback molecules like USP18. USP18 is a key negative regulator of IFN-I signaling, and its expression is elevated in fibrotic mouse models. Blocking IFNAR-1 can reduce macrophage numbers and alleviate fibrosis, partly by regulating the balance of STAT3/STAT1 phosphorylation (32). By enhancing such endogenous negative feedback mechanisms, excessive IFN-I responses can be more precisely downregulated, avoiding the immune deficiency risks associated with complete blockade. This provides a more selective intervention for reversing immune exhaustion.

### 5.2 Targeting downstream immune checkpoints

Immune checkpoint molecules induced by chronic IFN signaling are direct effectors of T cell exhaustion; therefore, targeting these downstream immunosuppressive molecules is a core strategy for reversing exhaustion. Immune checkpoint blockade therapies, such as using anti-PD-1, anti-TIGIT, and anti-LAG-3 antibodies, aim to directly block the inhibitory pathways induced by IFN signaling, restoring T cell effector functions (37).

In cancer treatment, immune checkpoint blockade has shown tremendous potential, and its principles are similarly applicable to immune exhaustion caused by chronic viral infections. Studies have shown that the expression of checkpoint molecules such as PD-1 in HIV-infected individuals correlates with the persistence of viral reservoirs. Therefore, ICB is being explored in combination with latency-reversing agents to reverse exhaustion and enhance the immune system’s ability to clear exposed reservoir cells (37). In this context, immune checkpoint inhibitors have been extensively investigated in people living with HIV. A comprehensive review highlighted that chronic HIV infection shares a similar state of T cell exhaustion with cancer, and that checkpoint blockade may not only restore HIV-specific T cell function but also facilitate reservoir reduction, although safety and efficacy in this population require further validation (14). In clinical practice, immune checkpoint inhibitors approved for various cancer types have demonstrated safety and efficacy in people living with HIV (PWH), and these patients should receive the same standard-of-care, full-dose cancer therapy as the general population unless data specific to HIV cohorts indicate otherwise. Moreover, prospective studies and an international retrospective analysis have shown that anti-PD-(L)1 agents are safe and effective across expected cancer types and CD4^+^ T cell counts, supporting their use in PWH for FDA-approved indications (58). Given that immune exhaustion is driven by multiple signaling pathways, combined blockade of multiple checkpoints may yield better results. Research shows that palmitoylation modification of TIM-3 can stabilize its expression, promote immune exhaustion, and weaken anti-tumor immunity. This suggests that targeting post-translational modifications of such checkpoint molecules may represent a new therapeutic strategy (59). Additionally, in the tumor microenvironment, IFN-I can induce PD-L1 expression on tumor cells and leukocytes. Blocking IFNAR can reduce this induction and promote anti-tumor T cell responses, indicating that combined targeting of the IFN signaling and the PD-1/PD-L1 axis may have a synergistic effect (60).

Therefore, developing combination therapies targeting multiple exhaustion-related molecules such as PD-1, TIGIT, LAG-3, and TIM-3, or combining them with drugs that modulate IFN signaling such as JAK inhibitors or IFNAR antibodies, may require combination strategies targeting multiple levels of immune dysfunction. This approach is expected to more effectively reverse deep immune exhaustion in the context of HIV-1 infection.

### 5.3 Combined reservoir-targeting strategies

Combining IFN signaling regulators or immune checkpoint blockers with latency-reversing agents constitutes a “shock and kill” strategy aimed at clearing the reservoir. The core logic is that LRAs, such as histone deacetylase inhibitors, activate latent viral reservoirs, inducing viral antigen expression so that they can be recognized by the immune system. Concurrently, the administration of ICB or IFN signaling regulators is used to reverse local T cell exhaustion that may be exacerbated by re-exposure to viral antigens, and to enhance the recognition and killing ability of immune cells such as cytotoxic T lymphocytes and natural killer cells against activated reservoir cells (61). For example, preclinical studies have shown that the combined use of CD4 mimetics with CD4-inducing antibodies can stabilize the conformation of the HIV-1 envelope protein, making it more recognizable by antibody-dependent cellular cytotoxicity, thus reducing viral rebound after interruption of combined antiretroviral therapy (61). Clinical evidence from a systematic review of immune checkpoint inhibitor use in people living with HIV demonstrated that, although most patients maintained stable viral loads and CD4^+^ T cell counts, a subset showed transient viral load increases followed by enhanced HIV-specific CD8^+^ T cell responses and decreases in cell-associated HIV-DNA, providing proof-of-concept for checkpoint blockade as a “shock-and-kill” strategy, albeit with modest overall efficacy (62).The challenge of this strategy lies in precisely controlling treatment timing and dosage. The activation by LRAs must be sufficient to expose the reservoir but not cause excessive systemic immune activation or cytokine storms, which could introduce toxicity and offset the benefits of immunotherapy (18, 37).

Furthermore, ensuring that the reinvigorated immune cells can effectively migrate to and eliminate the activated reservoir cells is crucial. A preprint study suggests that in tuberculosis infection models, excessive IFN-I signaling can impair the motility and surveillance function of CD8^+^ T cells at the lesion site. Blocking IFNAR can restore T cell motility (63). This suggests that in the “shock and kill” strategy, regulating the local IFN environment may be critical for the successful homing and killing of immune cells. Therefore, future research needs to optimize the combinations, administration sequences, and treatment durations of LRAs and immune modulators, and utilize specific immune activation or exhaustion markers as biomarkers to monitor treatment responses and toxicity, to achieve the ultimate goal of safely and effectively clearing viral reservoirs. Despite these promising strategies, several challenges remain, including tissue specificity, individual heterogeneity, and long-term safety, which we will discuss in the next section.

The bidirectional interplay between major HIV-1 curative strategies (e.g., “shock and kill” and “block and lock”) and the IFN-I signaling pathway, along with the resulting therapeutic implications, is summarized in **Table 1**. The key intervention nodes along the “reservoir-IFN-exhaustion” axis, along with their representative drugs, potential applications in curative strategies, advantages, and risks, are summarized in **Table 2**.

**Table 1:** Interplay between curative strategies and IFN signaling and therapeutic implications.

Curative strategy	Drug class	Representative agent(s)	Potential impact on IFN signaling	Potential impact on immune exhaustion	Mechanistic basis	Proposed combination
Shock and kill	Epigenetic latency-reversing agents	HDAC inhibitor-like approaches	May induce viral transcription and inflammatory gene programs in a context-dependent manner	May transiently increase exhaustion-associated signaling	Reservoir reactivation may alter local inflammatory tone	Consider combination with immune-modulatory strategies when excessive inflammation occurs
Shock and kill	Transcription-activating latency-reversing agent	PKC agonist-like approaches	May activate transcriptional and inflammatory signaling pathways	May promote T cell overactivation if not properly controlled	Potent transcriptional activation may amplify immune perturbation	Consider pathway-sparing immune modulation
Shock and kill	Transcriptional elongation-modulating agents	BET inhibitor-like approaches	May influence HIV transcriptional elongation and inflammatory programs	Effects on exhaustion-related signaling remain context-dependent	Transcriptional elongation can affect latency reversal and inflammatory output	Consider combination with immune-supportive strategies
Block and lock	Viral transcription-suppressing agents	Tat-targeting approaches	May reduce low-level viral RNA production	May indirectly reduce chronic inflammatory stimulation	Suppression of viral transcription may reduce innate immune sensing	Could be combined with IFN-pathway modulation in selected settings

**Table 2:** Comprehensive intervention strategies targeting the “reservoir-IFN-exhaustion” axis.

Target level	Target molecule	Intervention approach	Representative agent(s)	Application in curative strategies	Advantages	Risks	References
Upstream viral sensing	cGAS/STING pathway	Inhibitor	Experimental cGAS/STING inhibitors	May reduce DNA-sensing-driven IFN activation	Could lower chronic innate immune stimulation	May impair normal antiviral defense	(23–25)
IFN signaling	IFNAR	Receptor blockade	IFNAR-blocking approaches	May attenuate persistent IFN-I signaling	Directly interrupts IFN signaling cascade	Infection risk; uncertain HIV efficacy	(30, 53)
signal transduction	JAK/STAT pathway	Pathway inhibition	JAK-pathway inhibitors	May dampen downstream inflammatory signaling	Broadly suppresses IFN-associated transcriptional output	Opportunistic infection risk; off-target effects	(55, 56)
Downstream effector	PD-1 axis	Checkpoint blockade	Anti-PD-1-type approaches	May partially relieve inhibitory signaling	Directly targets exhaustion-associated inhibition	Immune-related adverse events; viral rebound concerns	(13, 15, 16, 37)
Downstream effector	TIM-3 axis	Checkpoint modulation	Anti-TIM-3-type or checkpoint-modulating approaches	May complement other checkpoint approaches	Potentially addresses multi-checkpoint exhaustion	Monotherapy efficacy may be limited	(13, 36, 37, 59)
Cellular metabolism	Mitochondrial fitness/metabolic flexibility	Metabolic modulators	May support T cell persistence and function	Addresses exhaustion-related bioenergetic defects	Improves T cell persistence and memory formation	HIV-specific therapeutic evidence remains limited	(40–43)

## 6 Challenges and future directions

### 6.1 Tissue reservoirs and local IFN microenvironments

Anatomical sites such as lymphoid tissue and intestinal mucosa are key ecological niches for the formation and maintenance of HIV-1 reservoirs. The local IFN signals in these tissue microenvironments may drive immune exhaustion more effectively than peripheral blood (64). Studies of the tumor microenvironment provide important references for understanding tissue-specific immune regulation. For example, in hepatocellular carcinoma and head and neck squamous cell carcinoma, there is significant heterogeneity in the tumor immune microenvironment across different anatomical sites with respect to T cell infiltration, expression of exhaustion markers such as PD-1, and patient prognosis (65). This suggests that HIV-1 may also shape unique local IFN microenvironments in different tissue reservoirs, such as gut-associated lymphoid tissue and the central nervous system, thereby affecting immune cell function in a tissue-specific manner.

Single-cell sequencing technology has been successfully applied to elucidate differences in the immune landscape of the liver and blood in patients with chronic hepatitis B, revealing unique transcriptional profiles and functional states of tissue-resident immune cells (66). Therefore, it is essential to develop and apply similar high-resolution *in situ* research techniques—such as single-cell multi-omics analysis of lymphoid tissue or intestinal mucosa biopsy samples—to accurately depict the dynamic interactions between viral transcriptional activity, local IFN responses (e.g., specific ISG expression profiles), and the functional states of immune cells, especially tissue-resident memory T cells and macrophages, in these hard-to-reach microenvironments (67, 68). Understanding this anatomical site-dependent heterogeneity is crucial for designing effective interventions. For instance, localized delivery strategies targeting the intestinal mucosa may need to differ from strategies targeting central nervous system reservoirs to achieve synergistic control of systemic reservoirs and immune pathology.

Future interventions should incorporate tissue-specific delivery systems. For example, using nanocarriers targeted to gut-homing receptors (e.g., integrin α4β7) to deliver IFNAR antagonists or JAK inhibitors directly to gut-associated lymphoid tissue could locally disrupt pathogenic IFN signaling while preserving systemic antiviral immunity. Additionally, a single-cell multi-omics-guided “shock and kill” strategy could be employed: identifying LRA-sensitive cell subsets within different tissue reservoirs via scRNA-seq of biopsy samples, while simultaneously assessing their accompanying IFN-ISG activation levels, thereby tailoring a combined “LRA + localized IFN-suppression” regimen for each anatomical site.

### 6.2 Individual heterogeneity and biomarkers

HIV-1-infected individuals display substantial inter-individual variability in multiple key parameters, including reservoir size and composition, type I IFN response intensity, and the degree of T cell exhaustion (69, 70). This heterogeneity partly stems from host genetic factors (e.g., HLA type), baseline immune status, co-infections, and differences in the gut microbiome (64). Therefore, identifying biomarkers that can predict individual responsiveness to immune interventions is vital.

In cancer research, biomarkers based on IFN-related gene signatures have been developed to predict prognosis and responses to immunotherapy. For instance, in colorectal adenocarcinoma and bladder cancer, an IFN-γ-related long non-coding RNA signature or the expression levels of interferon-induced transmembrane protein 3 (IFITM3) have been shown to correlate significantly with tumor immune microenvironment characteristics, patient survival, and responses to chemotherapy or immunotherapy (71, 72). Similarly, in HIV-1 research, developing biomarker panels based on specific ISG expression profiles—such as those reflecting features of chronic versus acute IFN signaling—as well as biomarkers including exhausted T cell subsets like CD8^+^ T cells with high expression of TOX and TIM-3, or monocyte/macrophage characteristics, may help distinguish patients who are more likely to benefit from IFN signaling-targeted therapies or combination immunotherapies (73, 74).

Furthermore, exploring how baseline immune status—such as the initial T cell repertoire and innate immune cell function—and host genetic background influence responses to IFN signaling modulation therapies (e.g., IFN receptor blockers or STING agonists) is key to achieving truly personalized treatment (75). For example, the expression of interferon regulatory factor 8 (IRF8) has been shown to correlate with the remodeling of the immune microenvironment in lung adenocarcinoma and enhanced responses to immunotherapy (75). suggesting that similar molecules may become predictive markers for personalized HIV-1 treatment.

Based on these biomarkers, a stratified treatment algorithm should be developed. For instance, patients could be classified into: (i) “High-IFN-Exhausted” (large reservoir, sustained high ISG, high PD-1/TOX) – preferentially receiving a combination of IFNAR blockade + immune checkpoint inhibitors (e.g., anti-PD-1) to reverse exhaustion; (ii) “Low-IFN-Latent” (small reservoir, low ISG) – treated with LRAs + low-dose IFN-enhancers to purge latent virus; (iii) “Mixed” – following a sequential strategy, with JAK inhibitors first to temper IFN signaling, followed by a “shock and kill” approach. Prospective clinical trials are needed to validate whether such biomarker-driven stratified therapy outperforms a uniform standard regimen.

### 6.3 Long-term safety and functional balance

The primary challenge of employing long-term suppression or modulation of the IFN-I pathway as a therapeutic strategy is the potential increased risk of opportunistic infections or the induction of autoimmune responses, given the critical physiological roles IFNs play in antiviral responses and immune surveillance (76).

The benefit-risk ratio of such interventions must be carefully evaluated in preclinical and clinical studies. For example, in cancer treatment, strategies aimed at activating IFN signaling, such as using STING agonists, must also balance their anti-tumor effects with the potential systemic inflammation they may induce (77). Future research should aim not to completely abrogate IFN function but to achieve a transition from “pathogenic” chronic IFN signaling to “protective” acute or moderate IFN signaling (44). This implies the need to develop interventions that can precisely regulate the spatiotemporal dynamics of IFN signaling. For instance, using bispecific antibodies to target and deliver IFN-β to specific cell types such as Clec9A^+^ dendritic cells has shown potential in preclinical models to reshape the tumor microenvironment and enhance anti-tumor immunity while possibly limiting systemic toxicity (78).

Similarly, in the context of HIV-1, delivering IFN signaling modulators locally or for a short duration—such as by targeting delivery of IFN receptor antagonists to lymphoid tissues, or sequentially combining them with antiretroviral therapies and other immunotherapies (e.g., checkpoint inhibitors)—may help reverse immune exhaustion while preserving the host’s fundamental defense capabilities against pathogens (79, 80). Additionally, long-term monitoring of infection events, autoantibody production, and immune reconstitution in patients receiving such novel therapies is crucial for ensuring treatment safety.

To achieve a functional cure, the following innovative combinations could be explored: (i) “Pulsed IFN suppression + shock and kill”: Administer an IFNAR blocker only within a brief window (e.g., 3–5 days) during LRA dosing, to transiently interrupt IFN-mediated checkpoint upregulation and enhance CTL function, while preserving IFN-mediated antiviral surveillance long-term. After the clearance window, the IFNAR blocker is withdrawn to restore baseline IFN levels. (ii) “Bispecific antibody-drug conjugates” (e.g., anti-CD4 × anti-IFNAR): In the “shock and kill” phase, deliver the IFN-suppressive agent only to the surface of CD4^+^ T cells (the reservoir), avoiding systemic immunosuppression. (iii) “IFN-switching” strategy: Use an IFN-β receptor-specific agonist instead of IFN-α to induce a distinct downstream transcriptional program (e.g., stronger antiviral ISGs and weaker immunosuppressive TOX/PD-1 induction), thereby limiting exhaustion while controlling the virus. These strategies require initial proof-of-concept in humanized mouse models or SIV models.

To synthesize the foregoing mechanistic discussion, **Table 3** offers an integrated view of the multi-layered immune dysregulation—spanning PRR sensing, IFN-I subtype heterogeneity, and checkpoint-driven T cell exhaustion. On this basis, **Table 4** presents a set of rationally designed combination strategies aimed at achieving a functional cure via precise IFN-I axis modulation, and **Table 5** provides a transparent assessment of the evidence base underpinning each major conclusion.

**Table 3:** Key layers of immune dysregulation in HIV-1 infection.

Category	Molecule/pathway	Expression/activity pattern in HIV-1 infection	Functional impact on reservoir/immune dysfunction	Therapeutic implications
Innate immune sensing	PRRs (TLR3/7/8/9, RIG-I, MDA5, PQBP1)	Acute phase: Markedly upregulated; recognize HIV-1 nucleic acids and drive IFN-I production. Chronic phase: TLR4/8 are broadly downregulated at mucosal sites (gut), associated with dysbiosis and microbial translocation.	Impaired innate sensing in chronic phase → failure of viral clearance → persistent low-grade inflammation.	Early ART can partially preserve PRR expression; targeting the PQBP1–cGAS axis may restore innate recognition.
Type I IFN subtype heterogeneity	IFN-α (13 subtypes)	Peripheral blood: Sustained and significantly elevated; drives broad ISG expression and CD4^+^ T cell immune activation.	Chronic IFN-α signaling → CD4^+^ T cell dysfunction and exhaustion; positively correlates with reservoir size.	Recombinant IFN-α2a has been used clinically; targeting the pDC/IFN-I axis is under exploration.
	IFN-β (single subtype)	Gut mucosa: Specifically upregulated (very low/undetectable in peripheral blood); induces an ISG profile distinct from that of IFN-α.	Drives chronic intestinal inflammation and impairs immune barrier; downregulation of IFN-β-specific ISGs is linked to impaired anti-inflammatory responses.	IFNAR blockade can restore ILC3 function and alleviate gut inflammation, supporting a “block rather than boost” strategy.
Inhibitory immune checkpoints	PD-1	Highly expressed on HIV-specific CD8^+^ and CD4^+^ T cells; enriched on latently infected CD4^+^ T cells.	Suppresses T-cell proliferation and effector functions (cytokine production, cytotoxicity); expression positively correlates with disease progression and reservoir size.	Anti-PD-1/PD-L1 antibodies are being evaluated for the “shock-and-kill” strategy.
	TIGIT	Highly expressed on CD8^+^ T cells; frequently co-expressed with PD-1 on latently infected CD4^+^ T cells.	Suppresses CD8^+^ T cell cytotoxic function; promotes HIV/SIV persistence in latently infected cells.	Anti-TIGIT antibodies, as part of combination immunotherapy, may synergize with anti-PD-1.
	LAG-3	Highly expressed on exhausted CD4^+^ and CD8^+^ T cells; co-expressed with PD-1 and TIGIT on reservoir cells.	Suppresses T cell proliferation and effector functions; synergizes with PD-1 to drive deep T cell exhaustion.	Anti-LAG-3 antibodies are already approved in oncology and may be repurposed for HIV immune restoration.

**Table 4:** Future combination strategies based on IFN signaling modulation.

Strategy name	Core mechanism	Target population	Example drug combination	Predicted advantage	Major risk	Preclinical validation recommendation	References
Pulsed IFN suppression plus shock and kill	Transiently attenuate IFN-I signaling during latency reversal	Individuals with high IFN/ISG and exhaustion signatures	LRA-like approach plus IFNAR blockade and checkpoint modulation	May reduce IFN-associated checkpoint induction during viral re-expression	Incorrect timing may impair antiviral clearance	Humanized mouse models and time-course studies	(30, 37, 53, 61)
Targeted delivery of IFN modulators	Deliver IFN-modulating agents to reservoir-enriched or inflamed tissues	Patients requiring localized immune modulation	Tissue-targeted IFN-modulating formulation	May reduce systemic immunosuppression	Delivery complexity and tissue specificity	Platform development and biodistribution studies	(60, 78)
IFNAR-biased signaling modulation	Shift chronic pathogenic IFN signaling toward more controlled immune activation	Patients with persistent IFN-related inflammation	IFN-pathway modulation plus immune-supportive strategy	May preserve antiviral protection while limiting exhaustion	Biological specificity remains uncertain	*In vitro* assays and animal validation	(8, 9, 44)
Biomarker-guided stratification	Match intervention intensity to reservoir burden, ISG signatures, and exhaustion markers	Candidate patients for immune intervention	Biomarker-guided selection of IFN-modulating or immune-supportive approaches	May improve benefit-risk ratio	Biomarker thresholds are not standardized	Prospective HIV cohort validation	(29, 35, 73)
Autophagy support plus IFN modulation	Reduce IFN-I-associated inflammation while supporting T cell function	Patients with chronic inflammatory phenotype	Autophagy induction plus checkpoint or latency-targeting strategy	May improve immune fitness and responsiveness	Long-term metabolic and immune effects	Preclinical HIV/SIV validation	(11, 37, 61)

**Table 5:** Evidence hierarchy for the major sections.

Key claim	Established in HIV-infected humans	Supported by animal/*in vitro* models	Extrapolated from other diseases
1. HIV-1 Reservoir-Driven Chronic IFN-I Signaling: From Persistent Activation to Functional Switch	Persistence of proviral DNA and low-level HIV transcription in ART-treated individuals (1, 3, 4, 19, 21); persistence of proviral DNA (1, 19, 20); Persistent immune activation and IFN/ISG-related transcriptional signatures have been reported in chronic HIV infection (6, 7, 31); acute phase: higher ISGs correlate with lower viral load (29); chronic phase: persistent immune activation (6)	cGAS/RIG‑I recognition of HIV products (23–25); Humanized mouse model shows IFN‑I impairs ILCs and drives CD4^+^ T cell exhaustion (10); DC‑T co‑culture demonstrates STING/IFN‑I‑dependent suppression (30); SIV macaques show persistent innate activation on ART (28)	-
2. Mechanisms of IFN‑I Signaling Driving T Cell Exhaustion	IFN‑α correlates with PD‑1/TIGIT (13); TB co‑infection increases exhaustion markers (36); impaired germinal centers/Tfh function (47)	*In vitro* T cell exhaustion models (40, 43); animal models of lymphoid disruption (46)	Cancer: IFN‑I binds PD-1 promoter (34); metabolic reprogramming (OXPHOS↓, glycolysis↑) from tumor immunology (39, 41, 42); autoimmune abnormalities (35)
3. The Reservoir‑IFN Axis: Clinical and Experimental Evidence	Immune dysfunction signatures persist despite ART (6)	STING‑dependent immune suppression by HIV oligonucleotides reversed by IFNAR blockade (30); rapamycin reduces IFN‑I‑mediated inflammation and improves T cell response (11); humanized mouse model of chronic HIV‑1 shows IFN‑I drives exhaustion (10)	-
4. Targeting IFN‑I Signaling to Reverse T Cell Exhaustion		JAK inhibitors reduce PD-1 *in vitro* (55); preclinical ICB studies reviewed (37); CD4 mimetics + antibodies reduce reservoir in SIV (61); IFNAR blockade restores T cell motility in TB model (preprint) (63)	Anifrolumab in lupus (53); JAK inhibitors in IBD (57); IFNAR blockade alleviates liver fibrosis (32); cancer ICB (anti‑PD‑1) (Direct HIV clinical evidence is limited; most interventions are supported by preclinical HIV/SIV models or extrapolated from autoimmune disease, cancer, and inflammatory disease)
5. Challenges and Future Directions	-	Single‑cell multi‑omics in chronic hepatitis B (66); glioblastoma IFN‑I studies (67)	Tumor microenvironment heterogeneity (65, 68); cancer prognostic signatures (IFN‑γ‑related lncRNA, IFITM3) (71, 72); STING agonist toxicity in cancer (77); bispecific Clec9A‑PD‑L1‑IFN in mice (78)

## 7 Conclusion

The HIV-1 reservoir constitutes a dynamic, immunologically active pathological core that harbors persistent proviral DNA and may produce low-level viral transcripts under certain conditions, thereby driving sustained IFN-I signaling and establishing a “reservoir-IFN-exhaustion” axis. Chronic IFN-I signaling may contribute to T cell exhaustion by promoting checkpoint-associated, metabolic, and tissue-level immune dysfunction. These mechanisms collectively impair reservoir clearance, explaining the incomplete immune recovery observed despite suppressive ART and the rapid viral rebound following treatment interruption.

Therapeutic strategies targeting this axis include upstream IFNAR blockade or JAK inhibitors, mid-to-downstream PD-1/PD-L1 checkpoint blockade, and combinatorial approaches with shock-and-kill regimens. However, clinical evidence remains mixed, with risks of autoimmune-like adverse effects and transient viral rebound. Immunotherapy alone cannot eliminate reservoirs within specific tissue microenvironments, and inter-individual heterogeneity significantly influences treatment responses. Future research must map tissue-specific reservoir networks using organoids, humanized models, and single-cell multi-omics; rigorously test sequential combination regimens integrating latency-reversing agents, immunomodulators, and therapeutic vaccines; and develop precise biomarkers to assess reservoir activity, immune exhaustion, and treatment outcomes. Future studies should integrate tissue-level reservoir mapping, immune profiling, and preclinical testing of sequential combination strategies.
